# The Earliest Fleshy Cone of *Ephedra* from the Early Cretaceous Yixian Formation of Northeast China

**DOI:** 10.1371/journal.pone.0053652

**Published:** 2013-01-14

**Authors:** Yong Yang, Qi Wang

**Affiliations:** State Key Laboratory of Systematic and Evolutionary Botany, Institute of Botany, Chinese Academy of Sciences, Beijing, P. R. China; The Pennsylvania State University, United States of America

## Abstract

Bracts of female cones of extant gymnosperm *Ephedra* (Joint fir) are either colorful and fleshy (section *Ephedra*), or dry-winged and membranous (section *Alatae*), or dry and coriaceous (section *Asarca*), which have played a crucial role in long-distance seed dispersal that is responsible for a wide distribution of the genus in semiarid and arid areas of Eurasia, North Africa, North America, and South America. Recent molecular systematic studies on *Ephedra* have suggested that the fleshy bracts in character evolution may be plesiomorphic relative to the dry, membranous and coriaceous bracts. However, little is known about when the fleshy bracts of *Ephedra* have made their debut in the geological past. Herein, we describe a novel, fleshy bract-bearing female cone macrofossil from the Early Cretaceous (ca. 120—125 Ma) Yixian Formation in Liaoning, northeastern China. This cone bears three ellipsoid seeds subtended by only one whorl of fleshy bracts. Each seed has a thin outer envelope and an inner integument that extends upward and passes through the opening of the outer envelope, forming a thin and straight micropylar tube. Such a syndrome shows the closest similarity to an extant triovulate species *Ephedra intermedia* in the section *Ephedra*, but the latter bears a whorl of terminal fertile bracts and more than one whorl of inferior sterile bracts, and a thick outer envelope. Hence, we establish a new fossil species *Ephedra carnosa*. Our discovery provides the first direct macrofossil evidence for the previous molecular systematics of *Ephedra*, implying that the origin of fleshy bracts in *Ephedra* should not have been later than that of the membranous and coriaceous bracts by at least the Early Cretaceous.

## Introduction

**Figure 1:**
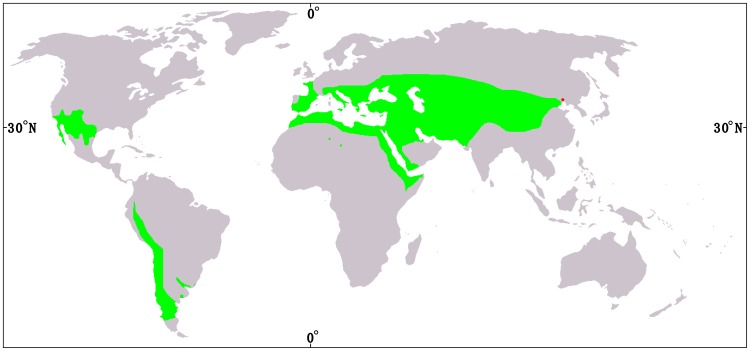
Distribution of extant *Ephedra* (green regions) after [**5**] (red dot showing the present fossil locality).

**Figure 2:**
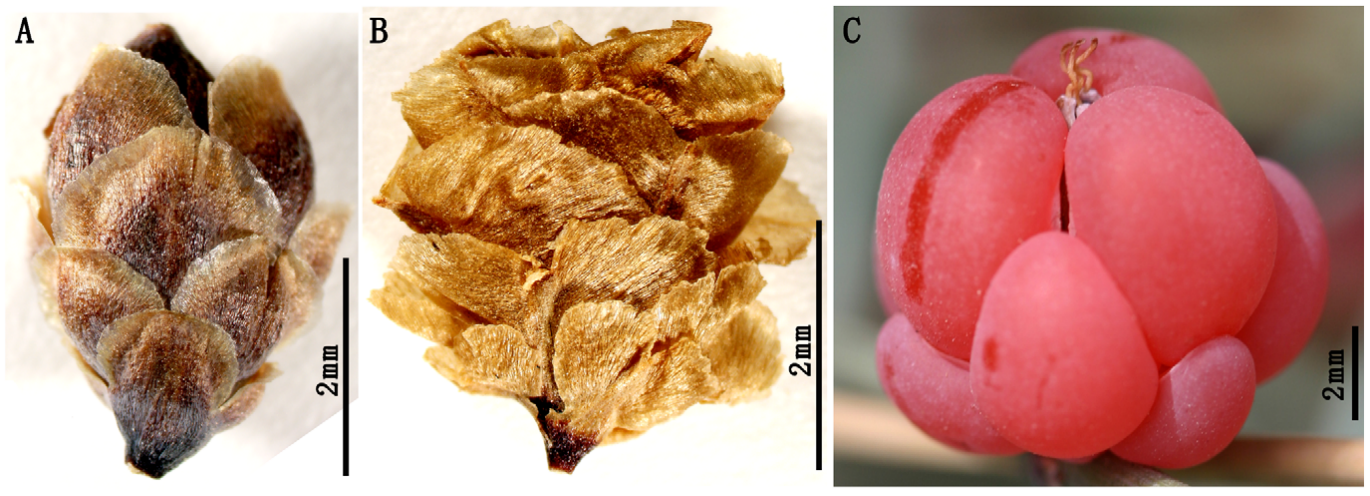
Representative female cones of three sections in *Ephedra*. A. A membranous female cone of *E. californica* Watson in Sect. *Alatae* Stapf. B. A coriaceous female cone of *E. strobilacea* Bunge in Sect. *Asarca* Stapf. C. A fleshy female cone of *E. intermedia* Schrenk et Mey. in Sect. *Ephedra*.

The gymnospermous genus *Ephedra* L. (Joint fir) contains about 50 living species, native to semiarid and arid areas of Asia, Europe, North Africa, North America, and South America ([1]–[7]; Fig. 1). This genus has three types of female cones upon which a once widely accepted sectional classification is based [8], i.e., Sect. *Alatae* Stapf bears free, dry, winged, and membranous bracts (Fig. 2A), Sect. *Asarca* Stapf has free, dry, but coriaceous bracts (Fig. 2B), while Sect. *Ephedra* possesses thickened, colorful, and fleshy bracts (Fig. 2C). Recent molecular phylogenetic studies have suggested that the three morphological sections are not natural and none of them is monophyletic, but the earliest diverged branch within the genus comprises species with fleshy cones from the Mediterranean region [9]–[12]. Meanwhile, Sect. *Alatae* and Sect. *Asarca* are nested within Sect. *Ephedra*, implying that the fleshy cone is plesiomorphic in *Ephedra*. However, when the first fleshy cone occurred in the fossil record remains unknown. *Ephedra* macrofossils (especially female cones) will provide an historical perspective for the early evolution, taxonomy, and biogeography of the genus.

Early *Ephedra* might have transformed bracts of female cones into fleshiness and vivid color to assist seed dispersal [13]–[15], resulting in a wide intercontinental distribution of the genus. However, little is known about when such characteristic fleshy cones occurred in *Ephedra*. According to molecular clock data, modern *Ephedra* was estimated to have a minimum age 8—32 Ma (i.e., an Oligocene origin) [11], [16]. However, it lags behind the first occurrence of Early Cretaceous meso- and macrofossils bearing clear morphology that can be reliably circumscribed within *Ephedra*, e.g., *E. archaeorhytidosperma* Yang et al. [17], *E. portugallica* Rydin et al., *E. drewriensis* Rydin et al. [18], and *E. hongtaoi* Wang et Zheng [19]. Therefore, pruning of early stem groups and extinctions of ancient lineages may have played an important role in the early evolution of *Ephedra* [11], [12], [20]. It is expected that the putatively plesiomorphic character bearing fleshy cones in *Ephedra* would have made their debut earlier than Oligocene.

In recent decades, numerous *Ephedra* and *Ephedra*-like meso- and macrofossils have been reported from the Early Cretaceous of South Europe, Northeast China, Mongolia, North America, and South America [17]–[19], [21]–[23]. Seed mesofossils with *in situ* pollen were reported from the Early Cretaceous of South Europe (Portugal) and North America [18], [24]. Macrofossils of reproductive shoots or female cones were found in the Early Cretaceous of South America [25], [26], and Mongolia [27], [28] and adjacent Northeast China [17], [19], [21]–[23], [29], [30]. Early Cretaceous strata of Northeast China contain a number of well-preserved ephedroid macrofossils that may shed light on the early evolution of *Ephedra*. They show high reproductive diversity but similar vegetative morphology, e.g., dichasial branching pattern (sometimes branches being clustered due to highly shortened internodes), long and linear leaves usually with two parallel veins, and internodes with longitudinal fine striations, which can be divided into three groups. The first group bears female cones with multiple whorls of fertile bracts, e.g. *Liaoxia* Cao et Wu ( = *Ephedrites* Göppert et Berendt) [21], [31]; the second group bears female cones with only one whorl of fertile bracts, e.g., *Ephedra hongtaoi* and *E. archaeorhytidosperma* [17], [19] and several species ascribed to *Gurvenella* Krassilov ( = *Chaoyangia* Duan) bearing trichotomous complex surrounding female cones [27], [32], [33]; the third group is *Siphonospermum* Rydin et Friis which has female cones without supporting bracts [22]. So far, all the previous studies have not provided any fossil evidence for the origin of fleshy female cones of *Ephedra*.

In this paper, we aim to describe a new, freshy cone-bearing fossil species, *Ephedra carnosa* Yang et Wang sp. nov., from the Early Cretaceous of Liaoning Province, Northeast China. Our discovery provides the first direct macrofossil evidence for the previous molecular systematics of *Ephedra*, implying that the origin of fleshy bracts in Sect. *Ephedra* should not have been later than that of the membranous and coriaceous bracts in Sect. *Alatae* and Sect. *Asarca* by at least the Early Cretaceous.

## Materials and Methods

**Figure 3:**
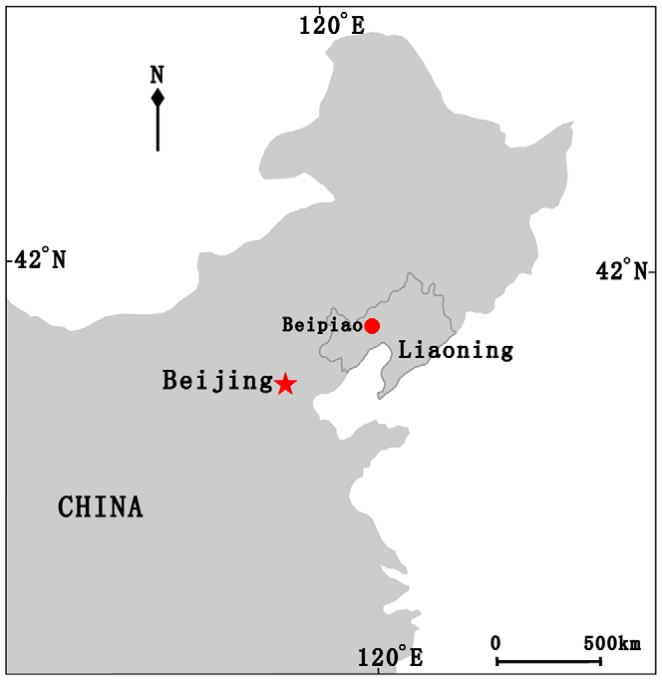
Fossil locality showing Beipiao (red dot) of Liaoning Province, Northeast China after [**40**].

The macrofossils used in this study were collected from the Yixian Formation at Huangbanjigou Village of Shangyuan Town, Beipiao City, Liaoning Province, Northeast China [Fig. 3]. The Yixian Formation is widely distributed in West Liaoning [33], and its geological age is the early Aptian—earliest late Aptian of the Early Cretaceous, which can be correlated by radiometric dating to about 120—125 Ma [34]–[39]. Previously, this formation has yielded a plethora of extraordinarily well-preserved freshwater and terrestrial fossils, especially including early angiosperms (e.g., *Archaefructus liaoningensis* Sun et al.), feathered theropod dinosaurs, early seed-eating birds, and primitive mammals [35]–[37], [39]–[41].

The macrofossils are preserved as impressions lacking cuticle and include a part and a counterpart from a gathering slab of finely laminated light grey to yellowish siltstone. Figures 1, 3 presented here were redrawn from the base maps, respectively [5], [40]. Voucher specimens (WH Lipsky 4181 and L Benson 15280) of *Ephedra californica* Watson and *E. strobilacea* Bunge were examined at the China National Herbarium (PE), including two female cones used in Figure 2A–B. The female cone of *Ephedra intermedia* Schrenk et Mey. presented in Figure 2C was photographed by the senior author at Mt Renshoushan of Gansu Province, Northwest China. Macrofossils were photographed with digital cameras (Nikon D700 and Panasonic DMC-FZ30) and under a microscope (Nikon Eclipse E600). Comparisons were made in Table 1 with some coeval, similar *Ephedra* and ephedroid meso- and macrofossils. All figures were arranged by Adobe Photoshop 6.0 and CorelDraw 10.0 programs. Terminology on the female cones of *Ephedra* [6], [42]–[44] used in the specimen descriptions was adopted. The specimens (including two gatherings PE 20120319A and 20120319B, 2012071006) are deposited at the China National Herbarium (PE), Institute of Botany, Chinese Academy of Sciences, Beijing, P. R. China.

**Table 1:** Key to extant and fossil species in *Ephedra* and other ephedroids.

1. Female cones lacking supporting bracts ------------------------------*Siphonospermum* [22]
–. Female cones bearing supporting bracts---------------------------------------------------------2
2. Female cones bearing multiple pairs/whorls of fertile bracts------------------*Liaoxia* [21]
–. Female cones bearing 1 pair/whorl of fertile bracts--------------------------------------------3
3. Female cones surrounded by furcated appendages-------------------------*Gurvanella* [27]
–. Female cones lacking furcated appendages------------------------------------------------------4
4. Cone bracts spine-like and lanceolate-----------------------------------------*Beipiaoa* [33], [54]
–. Cone bracts obtuse or acute when present-------------------------------------------------------5
5. Female cones bearing only a pair/whorl of terminal, fertile bracts and 1—multiple pair/whorls of inferior sterile bracts---------------------------------------------extant *Ephedra* [44]
–. Female cones having only a whorl of terminal, fertile bracts and lacking or having only a pair/whorl of inferior sterile bracts-------------------------------------------------- fossil *Ephedra* 6
6. Female cones having only a pair of inferior sterile bracts, and seed surface bearing transverse ridges------------------------------------------------------*E. archaeorhytidosperma* [17]
–. Female cones lacking inferior sterile bracts, and seed surface smooth--------------------7
7. Biovulate, and micropylar tube less than 0.8 mm-----------------------------*E. hongtaoi* [19]
–. Triovulate, and micropylar tube longer than 1 mm------------------*E. carnosa* [this paper]

### Nomenclature

The electronic version of this article in Portable Document Format (PDF) in a work with an ISSN or ISBN will represent a published work according to the International Code of Nomenclature for algae, fungi, and plants, and hence the new names contained in the electronic publication of a PLOS ONE article are effectively published under that Code from the electronic edition alone, so there is no longer any need to provide printed copies. The online version of this work is archived and available from the following digital repositories: PubMed Central, LOCKSS, BioOne.

## Results

The three extant gymnospermous genera, *Ephedra* L., *Gnetum* L., and *Welwitschia* Hooker, have been widely treated as a natural taxon, either a class Gnetopsida or an order Gnetales, with their own monogeneric families [3], [6], [7], [45], [46]. Therefore, our new fossil species of *Ephedra* is classified as follows:

Gnetopsida Eichler ex Kirpotenko, 1884

Gnetales Luerss., 1879

Ephedraceae Dumort., 1829

*Ephedra* L., 1753

**Figure 4:**
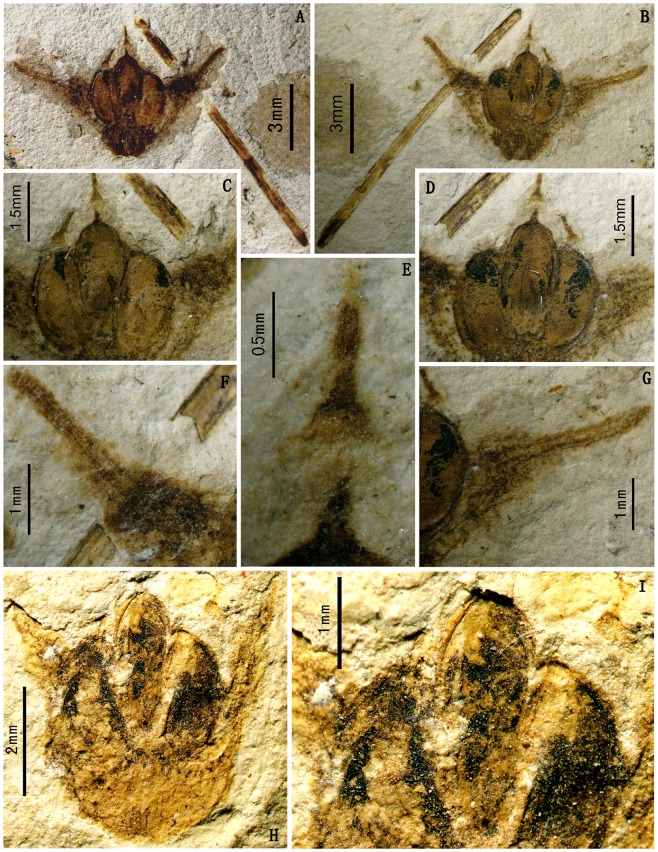
Fossil *Ephedra carnosa* Yang et Wang. A–B. A fleshy female cone and an associated axis. Holotype: PE 20120319A, B (part and counterpart). C–D. Enlargment of the female cone, showing morphology of three seeds. E. Close-up of the middle seed, showing a thin outer envelope and a straight micropylar tube. PE 20120319A. F–G. Close-up of the fleshy bract, bearing two veins sub-parallel in the middle-upper part and divergent toward the basal part. H. Another fleshy female cone. Paratype: PE 2012071006. I. Close-up of three seeds in Fig. H.

*Ephedra carnosa* Yang et Wang, sp. nov. (Fig. 4)

### Diagnosis

Triovulate cones have but only one whorl of bracts. Each bract is apparently thickened and spreading and subtends a seed. The bract bears two veins sub-parallel in the middle-upper part and divergent toward the basal part. Seeds are ellipsoid. The outer envelope is thin. The inner integument is fused with the nucellus, but only its apical part extends upward and passes through the outer envelope opening, forming a slim and straight micropylar tube.

### Description

The fossils presented here are triovulate cones, having but only one whorl of bracts [Fig. 4A–D, H–I]. A remnant receptacle is preserved at the bottom of the cone. The receptacle is 1.3 mm long and 1.3 mm wide at the base, and thickened acropetally up to 2.4 mm wide. The bracts are apparently thickened, spreading, triangular, and tapered [Fig. 4C–D, F–G], ca. 5—6 mm long and 3 mm wide at the base while 2 mm wide at the apex. The bract margin is not clearly defined. Each bract has two veins sub-parallel in the middle-upper part and divergent toward the basal part [Fig. 4F–G]. The interval between the two veins is wider at the base (up to 620 µm) and becomes narrower (ca. 207 µm) in the middle part, and then the two veins are parallel (ca. 138 µm) to each other towards the apex. Each bract subtends a seed. Seeds are ellipsoid, flat at the ventral side and arched at the dorsal side, about 1.5 mm wide and 2.6—3 mm long. The outer envelope is thin. The inner integument appears to be fused with the nucellus, but only its apical part extends upward and passes through the opening of the outer envelope to form a micropylar tube [Fig. 4E]. The micropylar tube is slim and straight, the exposed part is approximately1.3 mm long.

### Etymology

The specific epithet is derived from Latin “*carnosus*”, denoting the apparently thickened (thereby fleshy) bracts of female cones.

### Holotype

(Designated here)— PE 20120319A, B (part and counterpart), deposited at the China National Herbarium (PE), Institute of Botany, Chinese Academy of Sciences, Beijing, P. R. China.

### Paratype

PE 2012071006, deposited at the China National Herbarium (PE), Institute of Botany, Chinese Academy of Sciences, Beijing, P. R. China.

### Locality

Huangbanjigou Village of Shangyuan Town, Beipiao City, Liaoning Province, Northeast China.

### Stratigraphic horizon and age

Yixian Formation, the early Aptian—earliest late Aptian of the Early Cretaceous.

### Comparisons

The new fossil species *Ephedra carnosa* Yang et Wang is noticeably different from any other known extant and fossil species in *Ephedra* and other ephedroids [Table 1].

### Remarks

Bracts of female cones of *Ephedra* are modified foliar organs in nature. In living *Ephedra*, cone bracts have three states according to their mature morphology, e.g., fleshy bracts, dry and coriaceous bracts, and dry and membranous bracts (see Introduction, Fig. 2A–C). The thickness of median portion of cone bracts between the two parallel vascular bundles varies in the three sections of *Ephedra*, Sect. *Ephedra* (1.2—1.8 mm in *E. intermedia*), Sect. *Asarca* (ca. 80 µm in *E. californica*), and Sect. *Alatae* (ca. 40 µm in *E. torreyana*) (unpublished personal observations). This demonstrates that the fleshy cone bracts are markedly thicker than dry (either coriaceous or membranous) bracts in *Ephedra*. Similarly, the triovulate cones of our new macrofossil species *Ephedra carnosa* bear apparently thickened bracts without clearly defined margins, which are far more likely to be compared with those fleshy bracts in living *Ephedra*. In addition, two veins in each bract are sub-parallel in the middle-upper part (ca. 138—207 µm apart) but apparently divergent toward the basal part (up to 620 µm apart), implying that the bract would be most possibly swollen in life. Hence, the cone bracts of macrofossils *Ephedra carnosa* presented here are ripe and fleshy.

**Figure 5:**
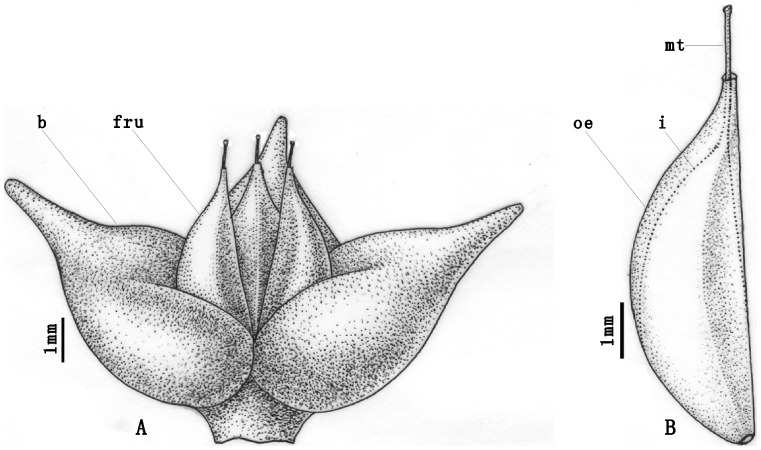
A schematic reconstruction of fossil *Ephedra carnosa* female cone and its seed. A. The triovulate cone of *E. carnosa*, bearing three spreading, fleshy bracts (b) and three female reproductive units (fru). B. The seed, showing the outer envelope (oe), inner integument (i) and a micropylar tube (mt).

The triovulate cones appear to have abscised from the reproductive shoots as a mature disseminule (or diaspore). Almost intact micropylar tubes in fossils imply that the cones have not been transported far from the parent plants before fossilization. On the basis of its trimerous nature, three seeds of each cone must be subtended by three verticillate bracts, which are 120 degrees distant from each other. Two bracts are visible, so the third bract might have run into the embedding rock after the cone fell into soft sediments, fossilized and then opened along the cutting plane. Building on the above morphological description and taphonomic inference, we present here a schematic reconstruction of the triovulate cone and its seed [Fig. 5].

There is an associated axis in the specimens besides the female cone described above. The axis is straight, 1.3 cm long and 0.4 mm wide, with fine longitudinal striations (Fig. 4A–B). These features imply that the axis is likely to be the vegetative twigs of a same parent plant (or population) as the female cone. Due to lacking organic connection and other useful epidermal evidence between them, we do not include the axis into the description of this new species.

## Discussion

### Characteristic thin outer envelope

The outer envelope of the new macrofossil species *Ephedra carnosa* seems very thin and vulnerable, so it is easy to be transformed after compression. Also, the integument extended into a micropylar tube enclosed in the outer envelope is visible. In contrast, modern *Ephedra* with fleshy bracts usually bears a thicker and harder outer envelope and a very thin integument with only 1—2-cell thick [44], [45]. In addition, Sect. *Alatae* usually bears thin outer envelope and integument while Sect. *Asarca* and Sect. *Ephedra* usually bear a thickened outer envelope. As a result, our new fossil species is quite unique in that it bears fleshy bracts and a very thin outer envelope.

The outer envelope of modern *Ephedra* has three types of ornamentations, including smooth surface (e.g., *E. intermedia*, *E. distachya* L., *E. aphylla* Forssk., and *E. tweediana* Fisch. et Mey.), papillate type (e.g., *E. major* Host, and *E. funerea* Coville et Morton), and transverse lamellar sculpture (e.g., *E. rhytidosperma* Pachomova, *E. trifurca* Torr. [6], [47], [48]. The smooth and/or striated surfaces are very common in both extant and fossil species while the other two types are only restricted to a few species. The transverse lamellar sculpture can also be found in fossil species from the Early Cretaceous [17], [49]–[51] and might have multiple origins [52]. Our new fossil species *Ephedra carnosa* bears the outer envelope with the smooth type of seed surface. Surface sculpture of the outer envelope may be variable in the developmental sequence. In both *E. equisetina* Bunge and *E. rhytidosperma*, the outer envelope is generally smooth at the early stages of development while specialized surface ornamentations only occur in the late stages of development [6]. In modern *Ephedra*, fleshiness is correlated with maturity of female cones. As a result, we infer that our new fossil species has ripe reproductive units with smooth outer envelope. Such a thin outer envelope may have some physiological functions, e.g., regulating water loss.

Two alternative hypotheses may be used to explain the thin outer envelope of this new fossil *Ephedra*. One is that this new species represents the stem lineage of *Ephedra* and it is the beginning of fleshy cones in response to animal (probably reptiles and birds) dispersal, and subsequent thickened outer envelope would have been evolved into the modern form; the other hypothesis is that the fossil species could not adapt to animal dispersal and become extinct because of its thin envelope. Hence it is an evolutionary blind alley of *Ephedra*.

### Ecological implications for the fleshy bracts

Three kinds of agents are known for the dispersal of *Ephedra*. The dry-winged, membranous bracts type of mature female cones is dispersed by wind while the coriaceous bract type is distributed by seed-catching rodents, and the fleshy bract type is dispersed by frugivorous birds [14], [15]. During Jurassic and Cretaceous, vertebrate-mediated (e.g., early mammals and early birds) seed dispersal interactions may have been important drivers of seed cone evolution in such conifers as Podocarpaceae Endl. and Taxaceae S. F. Grey, resulting in shifts of female cones from the lax open cones typical of Paleozoic and Triassic conifers to the more compact and reduced seed cones that are associated with fleshiness [53]. Remarkably, a seed-eating bird fossil has been discovered from the Yixian Formation of western Liaoning [39], [41]. Probably this is also the case in *Ephedra*, so fleshiness as an effective vertebrate-mediated seed dispersal mechanism may have accounted for the wide distribution of ephedroid plants in southern Europe, northeastern Asia, eastern North America, and South America during the Early Cretaceous.
