# Correction: The Earliest Fleshy Cone of *Ephedra* from the Early Cretaceous Yixian Formation of Northeast China

**DOI:** 10.1371/annotation/d1b28569-d9c0-4812-8332-9a6bfa9eb27f

**Published:** 2013-10-01

**Authors:** Yong Yang, Qi Wang

There were a number of errors in the legend for Figure 2, "Representative female cones of three sections in *Ephedra*," and in the second sentence of the Introduction. 

The complete, correct Figure 2 legend is: "A. A coriaceous female cone of *E. californica* Watson in Sect. *Asarca* Stapf. B. A membranous female cone of *E. strobilacea* Bunge in Sect. *Alatae* Stapf. C. A fleshy female cone of *E. intermedia* Schrenk et Mey. in Sect. *Ephedra*."

The correct second sentence in the introduction is: "This genus has three types of female cones upon which a once widely accepted sectional classification is based [8], i.e., Sect. *Asarca* Stapf bears free, dry, but coriaceous bracts (Fig. 2A), Sect. *Alatae* Stapf has free, dry, winged, and membranous bracts (Fig. 2B), while Sect. *Ephedra* possesses thickened, colorful, and fleshy bracts (Fig. 2C)." 

